# Quantifying the shear modulus of the adductor longus muscle during hip joint motion using shear wave elastography

**DOI:** 10.1038/s41598-023-36698-w

**Published:** 2023-06-12

**Authors:** Takuya Kato, Keigo Taniguchi, Taiki Kodesho, Gakuto Nakao, Yu Yokoyama, Yuhei Saito, Masaki Katayose

**Affiliations:** 1 Department of Physical Therapy, School of Health Sciences, Sapporo Medical University, Sapporo, Japan; 2 Research Fellow of Japan Society for the Promotion of Science, Tokyo, Japan; 3 Graduate School of Health Sciences, Sapporo Medical University, Sapporo, Japan

**Keywords:** Skeletal muscle, Ultrasonography

## Abstract

The present study aims to assess the effect of the hip flexion angle on the shear modulus of the adductor longus (AL) muscle associated with passive hip abduction and rotation. Sixteen men participated in the study. For the hip abduction task, the hip flexion angles used were − 20, 0, 20, 40, 60, and 80°, and the hip abduction angles were 0, 10, 20, 30, and 40°. For the hip rotation task, the hip flexion angles used were − 20, 0, 20, 40, 60, and 80°, hip abduction angles were 0 and 40°, and hip rotation angles were 20° internal rotation, 0° rotation, and 20° external rotation. The shear modulus at 20° extension was significantly higher than that at 80° flexion for the 10, 20, 30 and 40° hip abduction (i.e., *P* < 0.05). The shear modulus at 20° internal rotation and 20° extension was significantly higher than that at 0° rotation and 20° external rotation, regardless of the hip abduction angle (i.e., *P* < 0.05). The mechanical stress of the AL muscle associated with hip abduction was higher in the extended position. Furthermore, the mechanical stress could increase with internal rotation only at the hip-extended position.

## Introduction

When playing football, the hip adductor muscle is frequently injured. Some previous studies have reported that in football, the hip adductor muscle is the second most common injury site after the hamstring muscle^1,2^. Moreover, hip adductor muscle injuries are the most common cause of groin pain^3,4^, and among hip adductor muscles, the hip adductor longus (AL) muscle is the most frequently injured^5^. Although AL muscle injuries occur during kicking and changes in direction^5,6^, the mechanism of AL muscle injuries remains unclear.

To clarify the mechanism of AL muscle injuries, researchers have investigated the electromyography (EMG) of the AL muscle using surface EMG and observed changes in the muscle length of the AL muscle and hip joint angle using motion analysis and video analysis during soccer kicking and change of direction^7–11^, respectively. In particular, understanding the change in muscle length, which indicates AL muscle strain, is important for estimating the passive force that increases by muscle stretching^12^, causing muscle injury. Although changes in the AL muscle length associated with the hip joint angle have been reported^7^, the strain–stress (passive force) relationship of biological tissues is nonlinear^13,14^. Therefore, to elucidate the mechanism of AL muscle injuries, it is important to investigate how the changes in the passive force of the AL muscle are associated with the hip joint angle. Moreover, clarifying the changes in the passive force of the AL muscle is useful for developing the specific clinical examination for the AL muscle injuries and for effective stretching of the AL muscle.

Shear wave elastography (SWE) can quantify individual muscle tissue stiffness by measuring the muscle shear modulus, and it is used as a tool for indirect measurement of the mechanical properties of skeletal muscle, such as passive force^13,15^. We examined the shear modulus–passive force relationship in the AL muscle using SWE and demonstrated a strong linear relationship in each region of the AL muscle^16^. This result indicates that SWE can be used as an indirect measure of the passive force of the AL muscle. Based on this result, we revealed that the shear modulus of the AL muscle at 0° hip abduction depends on the hip flexion angle, whereas its increase depends on the hip extension^17^. In contrast, Ogawa et al.^18^ reported that the shear modulus of the AL muscle at maximum hip abduction is independent of the hip flexion angle. Although AL muscle injuries involve tri-planar hip motion, such as kicking and change of direction^6^, the effect of various hip motions that combine hip abduction, extension, and rotation on the passive force of the AL muscle has not been identified. Anatomically, the AL muscle constitutes a hip adduction moment arm and hip flexion, extension, and rotation moment arm^19^. Muscles with moment arms are subjected to passive forces during joint motion^20^. Hence, the increase in the passive force of the AL muscle depends on hip flexion, extension, and rotation, as well as hip abduction. Therefore, this study aimed to examine the changes in the passive force of the AL muscle at various hip positions by quantifying the shear modulus of the AL muscle using SWE. To accomplish this, we examined the effect of hip flexion angle on the shear modulus of the AL muscle associated with hip abduction and rotation. Based on previous findings that reported that the AL muscle constitutes a hip adduction, flexion, and internal rotation moment arm at the anatomical hip position (i.e., 0° hip flexion, abduction, and rotation)^19^, we hypothesized that the shear modulus of the AL muscle depends on the hip flexion angle, and its increase on hip abduction. Further, an increase in the shear modulus of the AL muscle associated with hip abduction and external rotation is higher in the hip-extended position.

## Methods

### Participants

Sixteen males (age, 26.3 ± 3.5 years; height, 173.9 ± 6.3 cm; weight, 69.4 ± 9.3 kg) with no history of neurological and orthopedical diseases in the trunk and lower limbs participated in this study. To perform repeated three-way ANOVA, a priori power analysis with an assumed type1 error of 0.05, a statistical power of 0.80, and an effect size of 0.40 was performed using G*power (3.1.9.6) analysis software. The minimum sample size was estimated as 11. Written informed consent was obtained from all the participants, and this study was approved by the Medical Research Ethics Committee at Sapporo Medical University (approval number: 1-2-25). All methods were performed in accordance with the relevant guidelines and regulations.

### Experimental procedure

**Figure 1:**
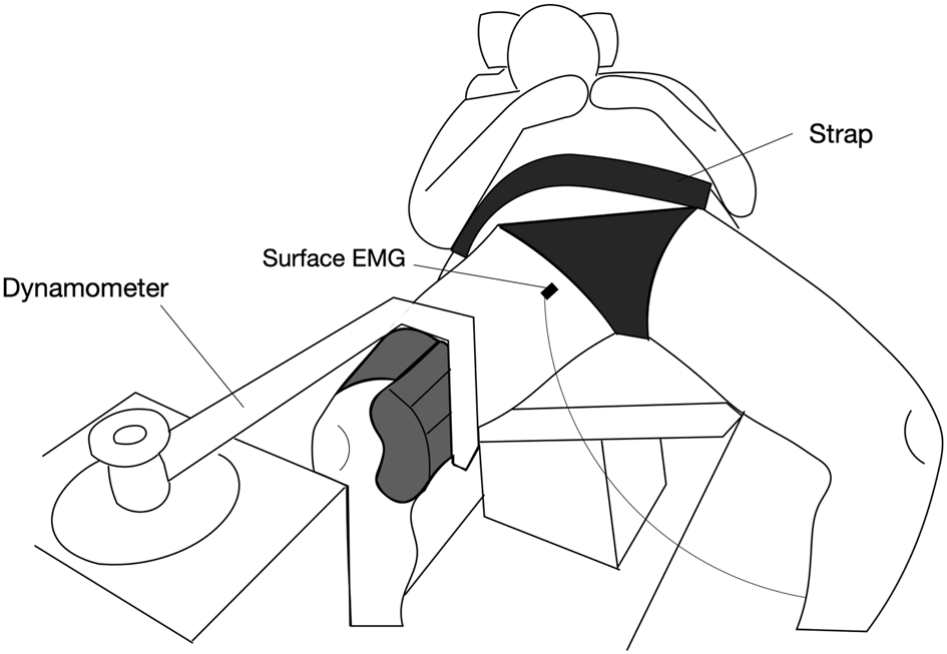
Experimental setup of the hip abduction task.

**Figure 2:**
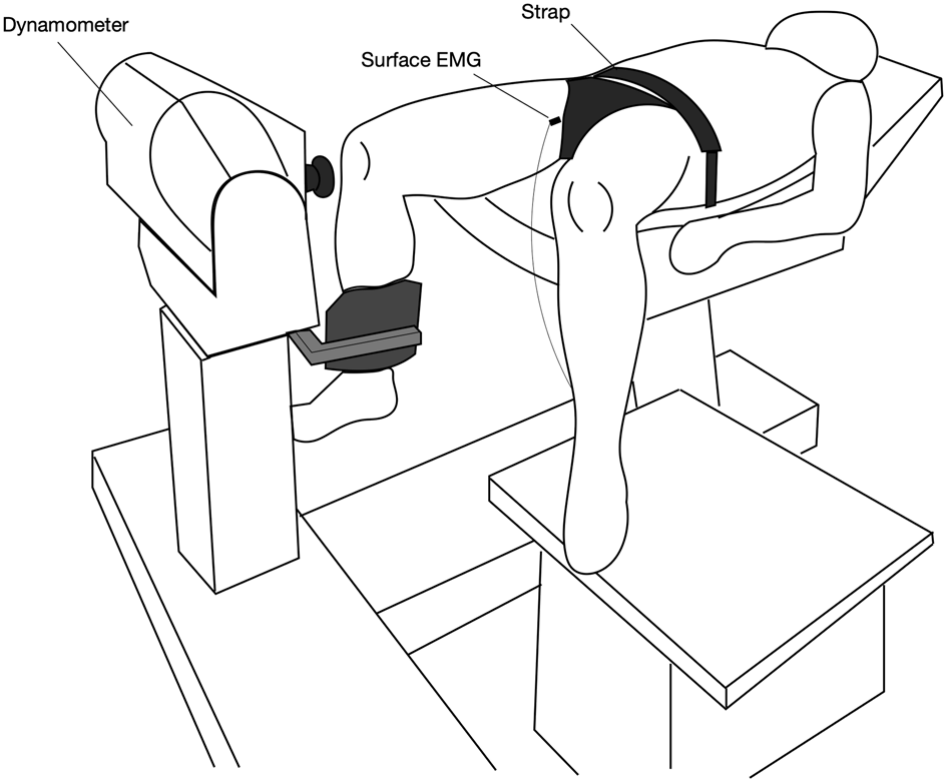
Experimental setup of the hip roration task.

The experiment in this study was conducted at a room temperature of 22 ℃ and humidity of 40%. We conducted two tasks: hip abduction and rotation. The first task examined the effect of the hip flexion angle on the shear modulus of the AL muscle associated with hip abduction, whereas the second task investigated the effect of the hip flexion angle on the shear modulus of the AL muscle associated with hip rotation. To reduce the effect of stretching on the shear modulus of the AL muscle, the two tasks were conducted on separate days spaced at least 48 h apart in a randomized order^21^. Hip abduction and rotation tasks were performed using an isokinetic dynamometer (Biodex System 4 Pro dynamometer, Biodex Medical System, Inc., Shirley, NY, USA) The participant was in a supine or sitting position, with the pelvis fixed to the seat with a strap. In the hip abduction task, the thigh was attached to the isokinetic dynamometer, and the distal end of the thigh was secured to the distal end of the force transducer (Fig. 1). The distance between the axis of the isokinetic dynamometer and the force transducer was set equal to that between the axis of the hip abduction/adduction and the force transducer. In the hip rotation task, the lower leg was attached to the isokinetic dynamometer, and the distal end of the lower leg was secured to the distal end of the force transducer (Fig. 2). The axis of the isokinetic dynamometer was aligned with axis of the hip rotation. The knee flexion angle was 90°. On the contralateral side, hip flexion, abduction, and rotation were set at 0, 20, and 0°, respectively. The knee flexion angle was set to 90°. In the hip abduction task, the shear modulus of the AL muscle was measured at hip abductions of 0, 10, 20, 30, and 40° at a hip extension of 20° (− 20° of hip flexion), hip flexions of 0, 20, 40, 60, and 80°, and hip rotation at 0°. The shear modulus of the AL muscle at the maximum hip abduction angle at each hip flexion angle was measured. The maximum hip abduction angle was defined at the onset of discomfort or pain^18^. The hip flexion angle was randomized, with hip abductions of 0, 10, 20, 30, 40°, and the maximum hip abduction angle. Hip flexions of 0, 20, 40, 60, and 80° were set by adjusting the angle of the dynamometer backrest. The hip extension angle was set to 20° by adjusting the height of the dynamometer seat. During the hip abduction task, passive hip adduction torque was measured using an isokinetic dynamometer. In the hip rotation task, the shear modulus of the AL muscle was measured at: 20° internal rotation; 0° of rotation; 20° external rotation at hip extensions of 20°; 0, 20, 40, 60, and 80° hip flexions; and 0 and 40° hip abductions. In addition to these hip positions, the shear modulus of the AL muscle was measured at the maximum internal and external rotation positions at each hip flexion angle and 40° hip abduction. The maximum internal and external rotation positions were defined at the onset of discomfort, pain, or trick movements, such as hip flexion, extension, abduction, and adduction. The hip flexion and rotation angles were randomized, and the order of the hip abduction angles was 0 and 40°. The hip flexion angle is defined as the angle formed between the line parallel to the center of the trunk and acromion and the line thigh segment marked by the anatomical points, greater trochanter, and lateral femoral epicondyle. The hip abduction angle is defined as the angle formed between the line perpendicular to the anterior superior iliac spine and the line equivalent to the long axis of the femur. Hip flexion and abduction angles were measured using a goniometer. The hip rotation angle is defined as the angle formed between the line perpendicular to the patella and the line equivalent to the long axis of the lower leg. For the hip abduction and rotation tasks, the hip rotation angles were measured using a goniometer and dynamometer, respectively. After the hip abduction and rotation tasks, isometric hip adduction tasks were performed to normalize the EMG. During the isometric hip adduction task, the participant was in the supine position on the dynamometer with the pelvis fixed to the seat using a strap. The participant performed two maximal voluntary contractions (MVC) of isometric hip adduction in a neutral hip position (0° hip flexion, abduction, and rotation) with manual resistance at the distal portion of the thigh. One minute of rest was allowed between the MVC tasks.

### Shear wave elastography

**Figure 3:**
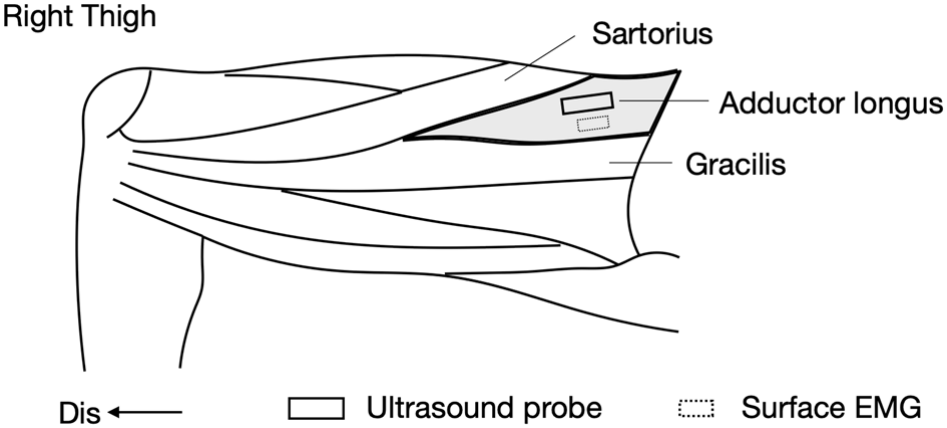
Location of the ultrasound probe and surface EMG electrode. *Dis* distal.

To measure the shear modulus of the AL muscle, SWE with a linear ultrasound transducer (2–10 MHz) (Aixplorer Ver.12 and SL 10-2; Supersonic Imagine, Aix-en-Provence, France) was used. To identify the measurement site of the shear modulus of the AL muscle, the probe was placed within 30% of the pubic symphysis and adductor tubercle of the femur (Fig. 3). The spaces between the AL and sartorius muscles and between the AL and gracilis muscles were marked on the skin with a pen using an ultrasonic B-mode image^17^. Subsequently, the position where the longitudinal image of the clearest fascicle of the AL muscle was obtained was determined as the measurement site of its shear modulus^17^. Previous studies demonstrated that the strong linear relationship between the passive force and shear modulus imaged in the long axis indicates that the probe position is parallel to the muscle; however, no such relationship existed between the passive force and shear modulus imaged in the short axis, indicating that the probe position is transverse to the muscle^22,23^. Moreover, our previous study demonstrated that the shear modulus of the AL muscle measured along the long axis was strongly linear (R^2^ = 0.98–0.99)^16^. Therefore, the probe was set along the long axis of the AL muscle.

SWE generates a shear wave within soft tissues. Based on the shear wave propagation velocity, *c*, the Young’s modulus, *E*, was quantified in kPa. *E* was mapped in the region of interest (ROI) using color. From each pixel of the ROI, *E* was calculated using *E* = 3ρc^3^, where density, *ρ*, was assumed to be constant (1,000 kg/m^3^) in human soft tissue^13^. The device used calculates *E* based on the assumption that the biological tissue is an isotropic material, whereas the muscle is anisotropic (i.e., its mechanical properties are not the same in all directions)^13^. Accordingly, we analyzed the shear modulus by dividing the obtained *E* by 3^24^.

### Electromyogram

A surface EMG of the AL muscle was recorded to confirm whether the AL muscle contraction occurred during hip abduction and rotation tasks. Surface EMG data were collected from the AL muscle using an active electrode (DE-2.1; Main Amplifier Unit, Bagnoli-8; Delsys, Boston, MA, USA). The electrode specifications in this study were as follows: frequency response, 20 ± 5 to 450 ± 50 Hz; differential amplification; 10 mm inter-electrode distance; two 1 mm × 10 mm silver bars as contact sensors; tenfold pre-amplifier gain; 1015 Ω/0.2 pF input impedance; 92 dB common mode rejection ratio; and 1000-fold main amplifier unit. The EMG signals were sampled at 1000 Hz and stored on a computer. The surface electrode of the AL muscle was attached parallel to the ultrasound probe, which did not overlap with the probe, and was confirmed to be within the area of the AL muscle using ultrasonic B-mode imaging (Fig. 3). Prior to the electrode attachment, the skin was shaved, abraded, and cleaned with a disinfectant.

### Data analysis

**Figure 4:**
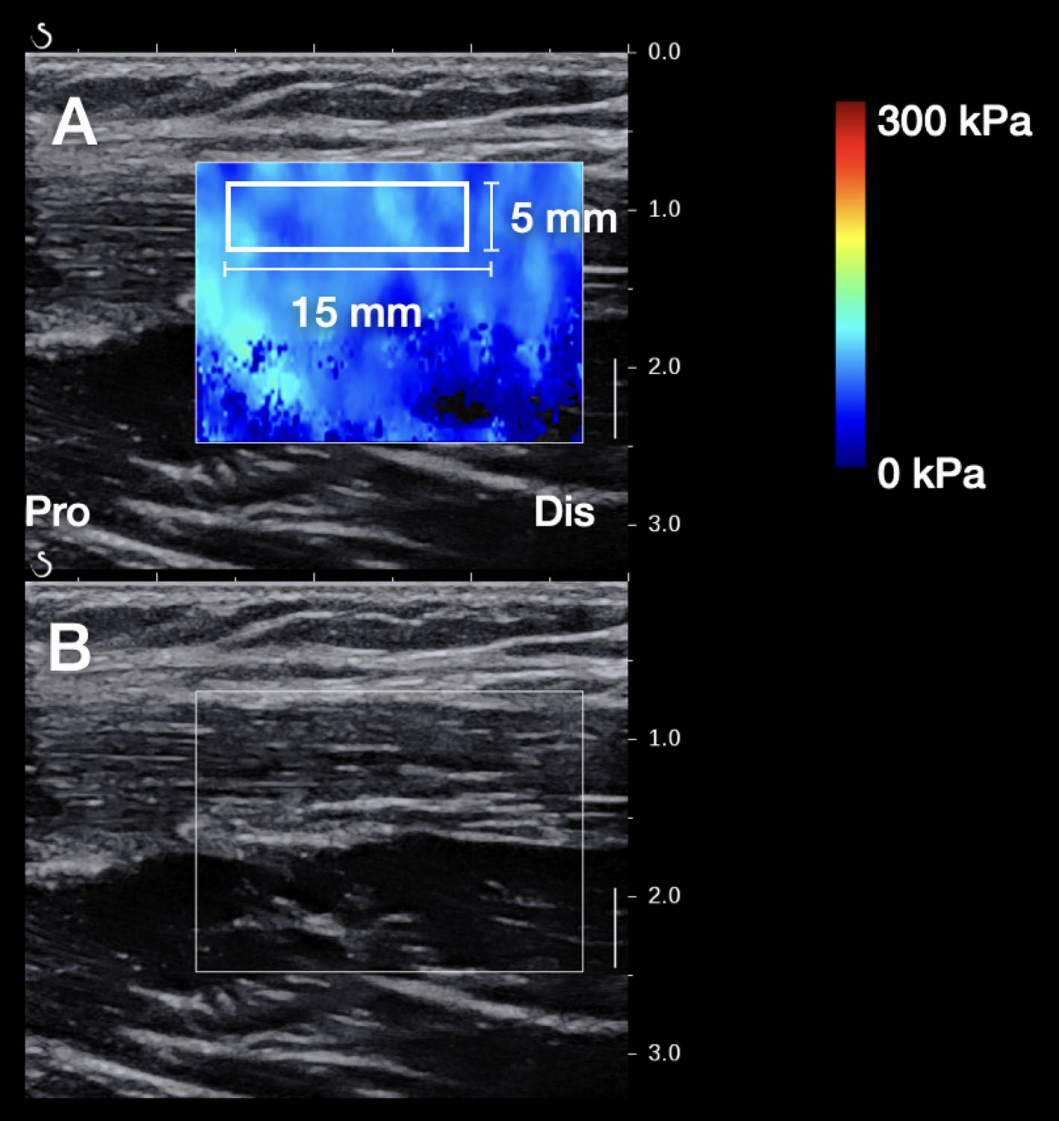
Typical examples of the SWE image (**A**) and B-mode image (**B**). *SWE* shear wave elastography, *Pro* proxima, *Dis* Distal.

The SWE data were exported as elasticity images in the JPEG format and analyzed using custom analysis software (S-14133 Ver.1.1, Takei Scientific Instrument Co., Ltd., Niigata, Japan)^17^. An ROI (width, 15 mm; height, 5 mm) that included a clearer fascicle without obvious connective tissue, such as the proximal intramuscular tendon, was marked on the color map (Fig. 4). The mean value of the shear modulus within the ROI was calculated. Subsequently, the average of two shear-modulus measurements for each hip position was used for statistical analysis.

EMG, passive hip adduction torque during the hip abduction task, and hip rotation angle during the hip rotation task were sampled at 1000 Hz using an analog/digital converter (Power Lab ML880, AD Instruments, Bella Vista, NSW, Australia) and stored on a computer using Chart software (Lab Chart 8, Ver 8.1.17, AD Instruments, Bella Vista, NSW, Australia). The EMG signals were high-pass filtered at a cut-off frequency of 20 Hz. To calculate the root mean square (RMS) for each session, the EMG signals were sampled at 200 ms across all hip positions without the pulse noise generated by SWE (frequency of occurrence: approximately 1 Hz). The RMS values during each hip position were normalized to the RMS value during MVC in the isometric hip adduction task. The passive hip adduction torque during the hip abduction task and hip rotation angle during the hip rotation task were sampled at 1 s and averaged for each hip position.

### Statistics

Data were statistically analyzed using SPSS (SPSS Statistics Ver.27.0; IBM, Armonk, USA). For each of the hip abduction and rotation tasks, intraclass correlation coefficient (ICC) estimates were calculated based on a mean-rating (k = 2), absolute-agreement, two-way mixed-effect model to evaluate the test–retest reliability of the SWE measurement. In addition, the coefficients of variance (CV) and standard error of measurement (SEM) were evaluated. Normal distributions consistently passed the Shapiro–Wilk test used to test normality. For the hip abduction task, the shear modulus data of the AL muscle, except those obtained from the maximum hip abduction angle, were analyzed using a two-way (flexion × abduction) ANOVA with repeated measures. The shear modulus of the AL muscle at maximal hip abduction was analyzed using one-way (flexion) ANOVA with repeated measures. For the hip rotation task, the shear modulus data of the AL muscle, except that obtained from maximum hip internal and external rotation angles, were analyzed using a three-way (flexion × rotation × abduction) ANOVA with repeated measures. The shear modulus data of the AL muscle at the maximum hip internal and external rotation angles with 40° hip abduction were analyzed using a two-way (flexion × rotation) ANOVA with repeated measures. Where relevant, post hoc tests (Bonferroni method) were conducted. Before ANOVA, Mauchly’s test of sphericity was applied, and if violated, the Greenhouse–Geisser correction factor was used to control a type 1 error risk. For the hip abduction task, the passive hip adduction torque was analyzed using one-way ANOVA (flexion) with repeated measures. Where relevant, post hoc tests (Bonferroni method) were conducted. The shear modulus of the AL muscle-passive hip adduction torque relationship for each hip flexion angle was analyzed using the Pearson product-moment correlation coefficient (r). The maximum hip abduction and internal and external rotation angles were analyzed using one-way ANOVA (flexion) with repeated measures. The level of statistical significance for all comparisons was set at *P* = 0.05.

## Results

### Measurement repeatability of shear modulus and EMG

The test–retest reliability of the SWE measurements was high in the hip abduction task for each hip flexion angle, respectively (Table 1). Similarly, it was also high in the hip rotation task for each hip flexion angle (Table 2).

**Table 1:** Test–retest reliability of SWE measurements in the hip abduction task.

Hip flexion angle	ICC (95% CI)	CV (%)	SEM (kPa)
− 20°	0.999 (0.999–1.000)	1.8 ± 1.5	1.06
0°	0.999 (0.999–0.999)	1.7 ± 1.4	0.89
20°	0.999 (0.999–0.999)	1.6 ± 1.5	0.86
40°	0.999 (0.998–0.999)	1.8 ± 1.8	0.66
60°	0.999 (0.999–1.000)	1.8 ± 1.6	0.50
80°	0.998 (0.996–0.998)	2.2 ± 2.3	0.58

*SWE* Shear wave elastography, *ICC* intraclass correlation coefficient, *95% CI* 95% confidence interval, *CV* coefficients of variance, *SEM* standard error of measurement.

**Table 2:** Test–retest reliability of SWE measurements in the hip rotation task.

Hip flexion angle	ICC (95% CI)	CV (%)	SEM (kPa)
− 20°	0.996 (0.994–0.997)	2.2 ± 2.1	0.55
0°	0.997 (0.995–0.997)	1.7 ± 1.6	0.38
20°	0.996 (0.995–0.997)	2.0 ± 2.2	0.39
40°	0.996 (0.995–0.997)	2.3 ± 2.1	0.44
60°	0.997 (0.995–0.998)	2.3 ± 2.2	0.33
80°	0.993 (0.991–0.995)	2.3 ± 2.7	0.35

*SWE* Shear wave elastography, *ICC* intraclass correlation coefficient, *95% CI* 95% confidence interval, *CV* coefficients of variance, *SEM* standard error of measurement.

The EMG readings (%RMS) of the AL muscle during the hip abduction and rotation tasks were 1.4 ± 0.8% and 1.7 ± 0.8%, respectively. There was no significant difference in the %RMS between the hip flexion angles in each task (hip abduction task: F = 0.84; *P* = 0.49; hip rotation task: F = 0.64; *P* = 0.59).

### Effect of hip flexion angle on changes in shear modulus of the AL muscle associated with hip abduction

**Figure 5:**
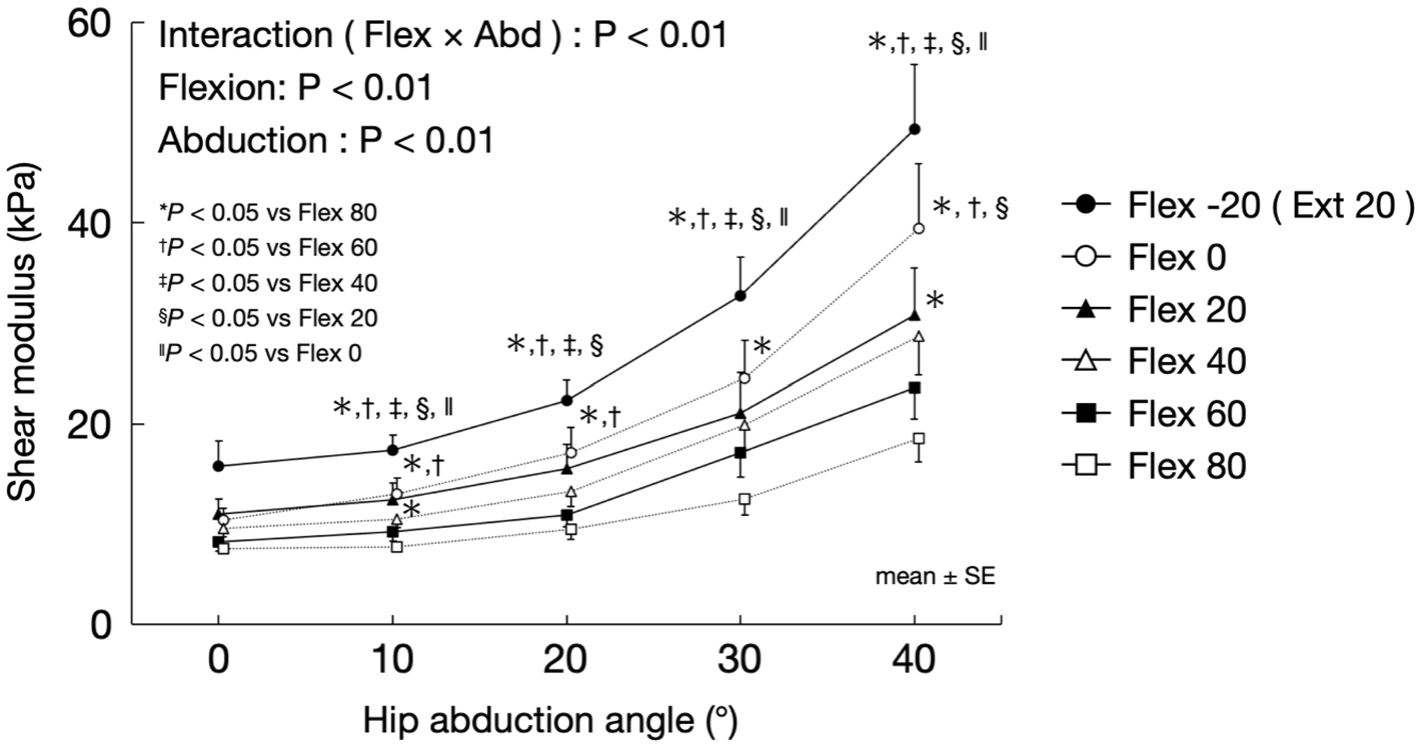
Changes in the shear modulus of the AL muscle associated with hip abduction in each hip flexion angle. *AL* adductor longus, *Flex* flexion, *Abd* abduction.

Figure 5 shows the changes in the shear modulus of the AL muscle associated with hip abduction for each hip flexion angle. A significant flexion-abduction interaction (F = 9.12, *P* < 0.01) was observed. The shear modulus of the AL muscle at the 20° hip extension was significantly higher than that at the 20, 40, 60, and 80° hip flexions for the 10, 20, 30 and 40° hip abduction and that at the 0° hip flexion for the 10, 30, and 40° hip abductions. The shear modulus of the AL muscle at the 0° hip flexion was significantly higher than that at the 80° hip flexion at 10, 20, 30, and 40° hip abductions, 60° hip flexion at 10, 20, and 40° hip abductions, and 20° hip flexion at 40° hip abduction. The shear modulus of the AL muscle at 20° hip flexion was significantly higher than that at 80° hip flexion angle at the 40° hip abduction. The shear modulus of the AL muscle at 40° hip flexion was significantly higher than that at 80° hip flexion angle at 10° hip abduction.

### Effect of hip flexion angle on the shear modulus of the AL muscle at the maximum hip abduction position

**Figure 6:**
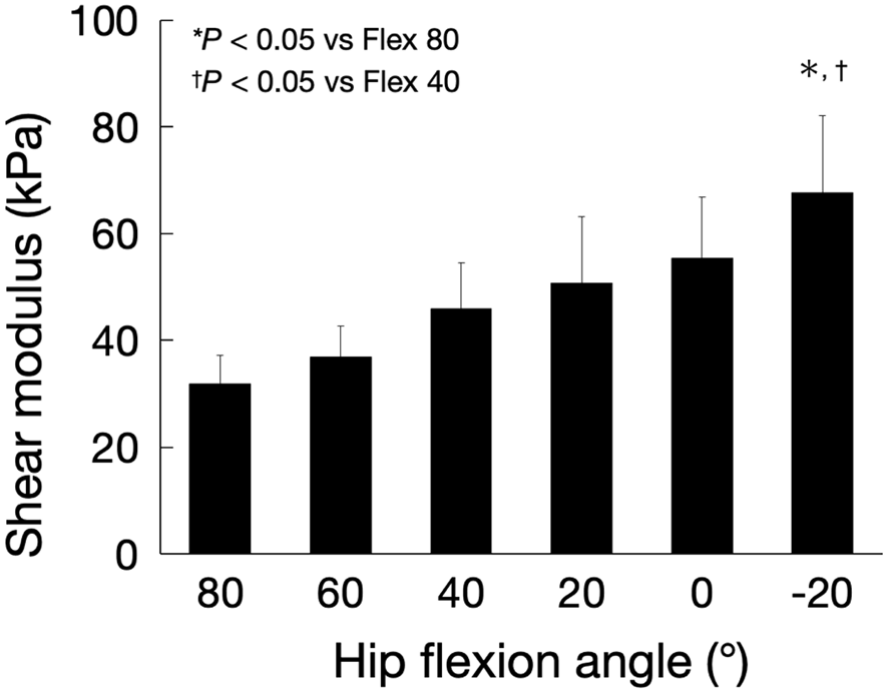
Shear modulus of the AL muscle at the maximum hip abduction angle for each hip flexion angle. *AL* adductor longus.

Figure 6 shows the shear modulus of the AL muscle at the maximum hip abduction angle for each hip flexion angle. ANOVA revealed a significant flexion effect (F = 8.09, *P* < 0.01). The shear modulus of the AL muscle at the 20° hip extension was significantly higher than that at the 40 and 80° hip flexions.

### Effect of the hip flexion angle on the changes in the shear modulus of the AL muscle associated with hip rotation

**Figure 7:**
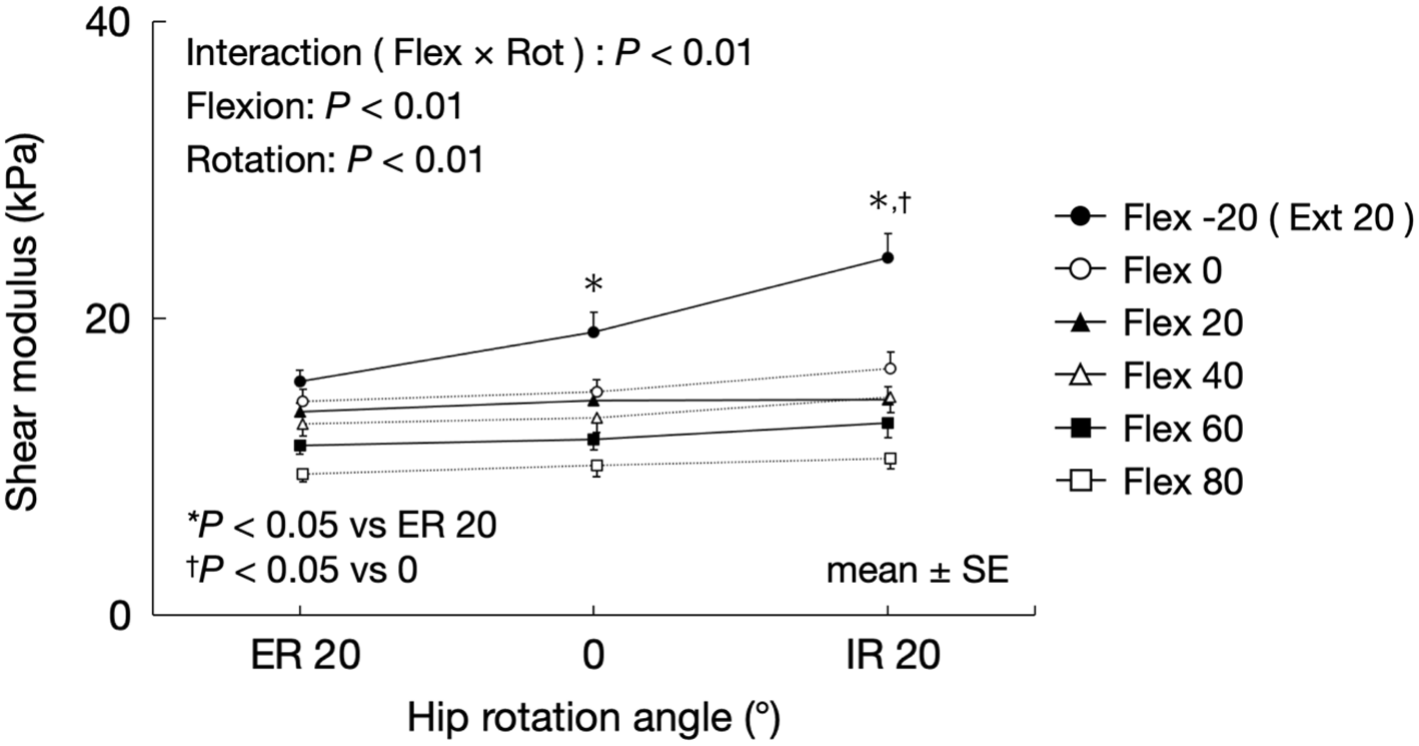
Changes in the shear modulus of the AL muscle associated with hip rotation for each hip flexion angle. *AL* adductor longus, *Flex* flexion, *Rot* rotation, *ER* external rotation, *IR* internal rotation.

Figure 7 shows changes in the shear modulus of the AL muscle associated with hip rotation for each hip flexion angle. According to a three-way ANOVA, there were significant flexion × rotation interactions (F = 9.63, *P* < 0.01) and flexion × abduction interactions (F = 6.26, *P* < 0.01). However, there were no significant flexion × rotation × abduction interactions (F = 1.98, *P* = 0.09) and rotation × abduction interactions (F = 1.19, *P* = 0.32). For the 20° hip extension, the shear modulus of the AL muscle at 20° hip internal rotation was significantly higher than that at 0° hip rotation and 20° hip external rotation. Moreover, the shear modulus at 0° hip rotation was significantly higher than that at 20° hip external rotation.

### Effect of hip flexion angle on shear modulus of AL muscle at the maximum hip rotation position

**Figure 8:**
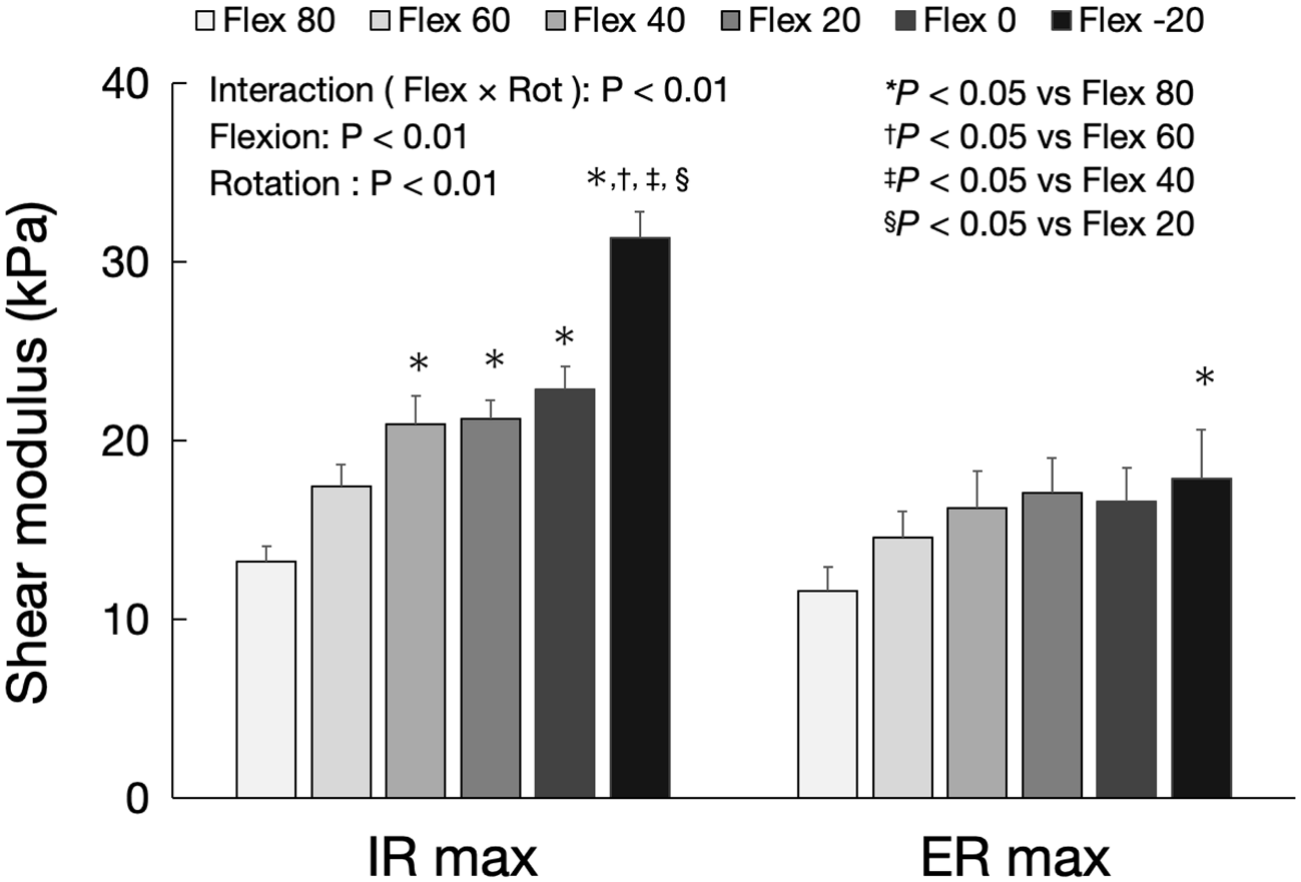
Shear modulus of the AL muscle at the maximum hip external and internal rotation angles for each hip flexion angle. *AL* adductor longus, *Flex* flexion, *Rot* rotation, *ER max* maximum external rotation, *IR max* maximum internal rotation.

Figure 8 shows the shear modulus of the AL muscle at the maximum hip external and internal rotation angles for each hip flexion angle. A significant flexion × rotation interaction (F = 7.18, *P* < 0.01), and significant effects of flexion (F = 16.52, *P* < 0.01) and rotation (F = 20.39, *P* < 0.01) were observed. At maximum hip internal rotation, the shear modulus of the AL muscle at 20° hip extension was significantly higher than that at 20, 40, 60, and 80° hip flexions (*P* < 0.01), and the shear modulus at 0, 20, and 40° hip extensions was significantly higher than that at 80° hip flexion (*P* < 0.01). At the maximum hip external rotation, the shear modulus of the AL muscle at 20° hip extension was significantly higher than that at 80° hip flexion (*P* < 0.01).

### Effect of hip flexion angle on passive hip adduction torque associated with hip abduction

**Figure 9:**
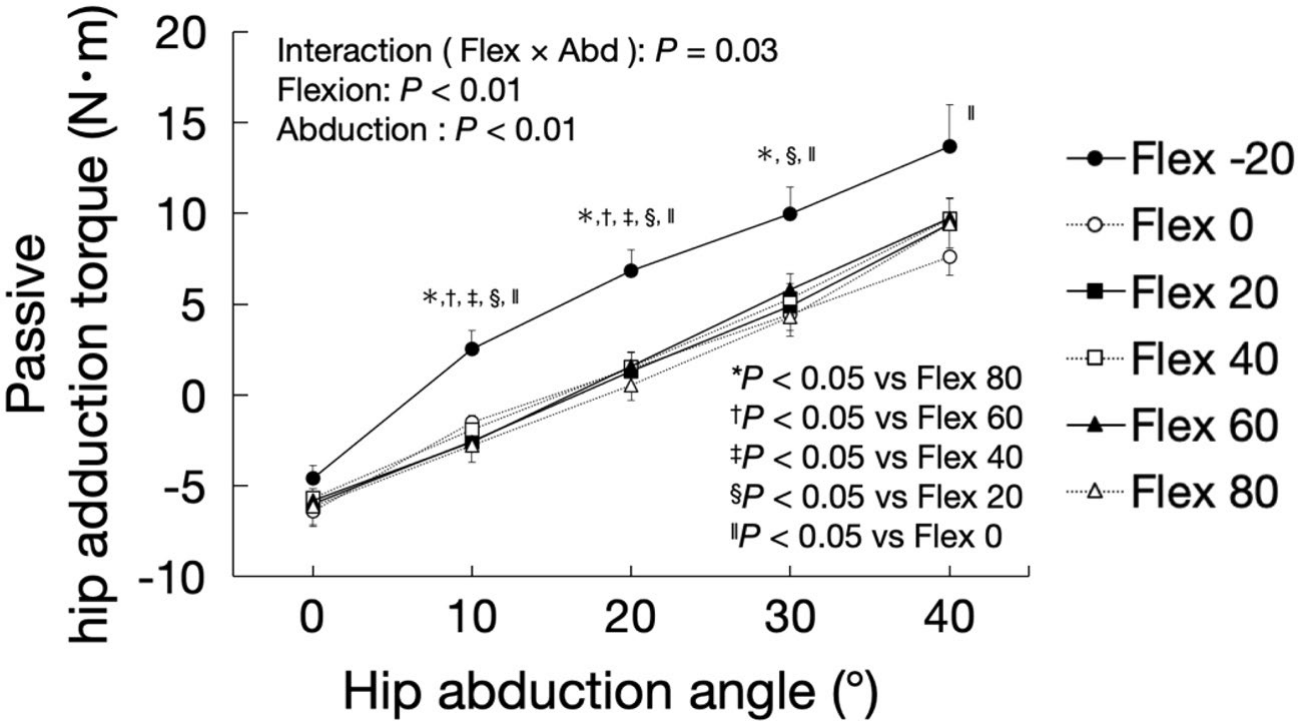
Changes in passive hip adduction torque associated with hip abduction for each hip flexion angle. *Flex* flexion, *Abd* abduction.

Figure 9 shows changes in passive hip adduction torque associated with hip abduction for each hip flexion angle. The significant flexion × abduction interaction (F = 2.45, *P* = 0.03) and effects of flexion (F = 12.40, *P* < 0.01) and abduction (F = 240.11, *P* < 0.01) were observed. The passive hip abduction torque at the 20° hip extension was significantly higher than that for 10 and 20° hip abductions, that at 0, 20, and 80° hip flexions for 30° hip abduction, and that at the 0° hip flexion for 40° hip abduction.

### Relationship between shear modulus of the AL muscle and passive hip adduction torque at each hip flexion angle

**Figure 10:**
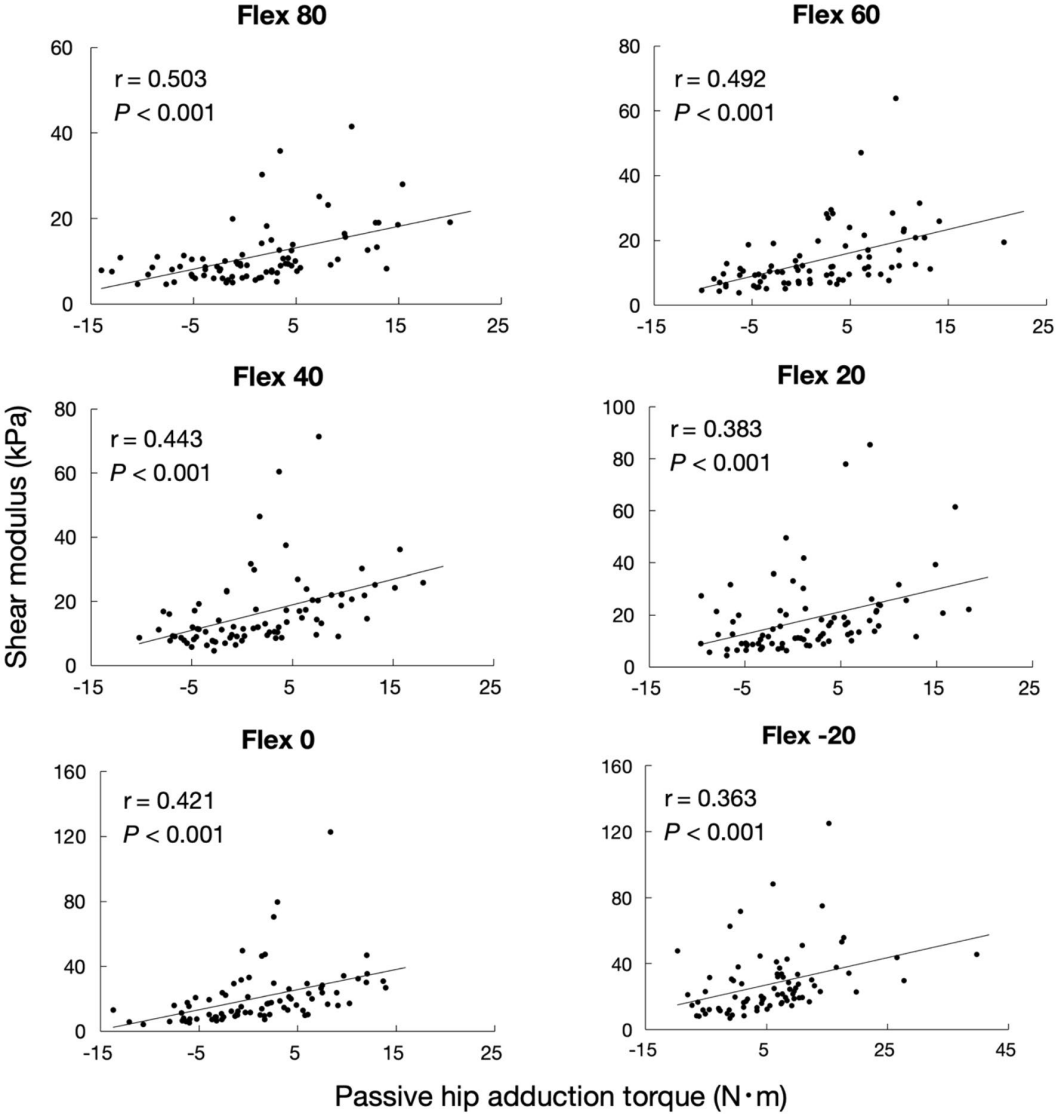
Relationship between shear modulus of the AL muscle and passive hip adduction torque for each hip flexion angle. *AL* adductor longus, *Flex* flexion.

Figure 10 shows the relationship between the shear modulus of the AL muscle and passive hip adduction torque for each hip flexion angle. The shear modulus of the AL muscle was significantly correlated with the passive hip adduction torque, regardless of the hip flexion angle (*P* < 0.01). The correlation coefficients (r) were 0.363, 0.421, 0.383, 0.443, 0.492, and 0.503 for 20° hip extension, 0, 20, 40, 60, and 80° hip flexions, respectively.

### Effect of hip flexion angle on the maximum hip abduction and internal and external rotation angle

**Figure 11:**
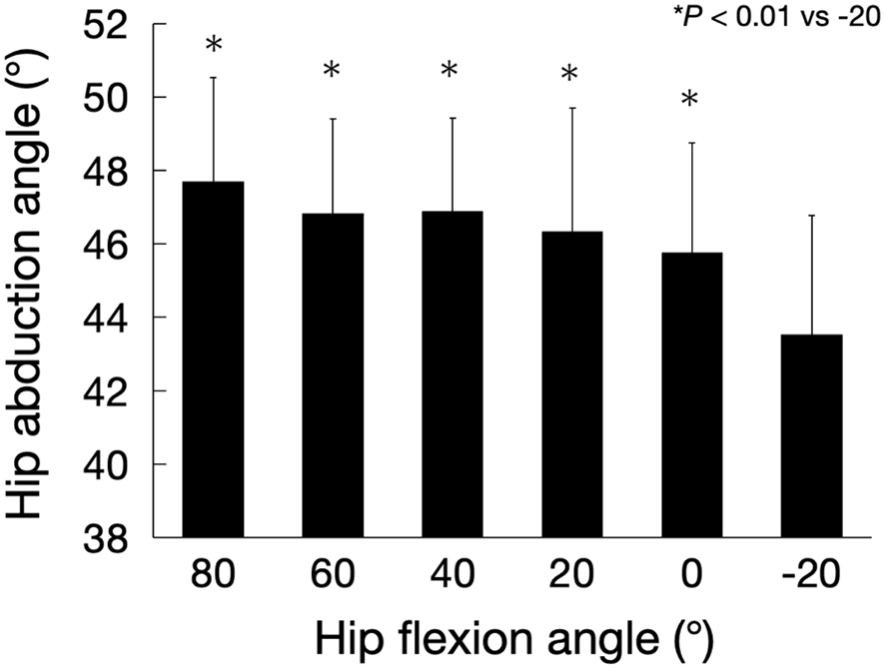
Maximum hip abduction angle for each hip flexion angle.

**Figure 12:**
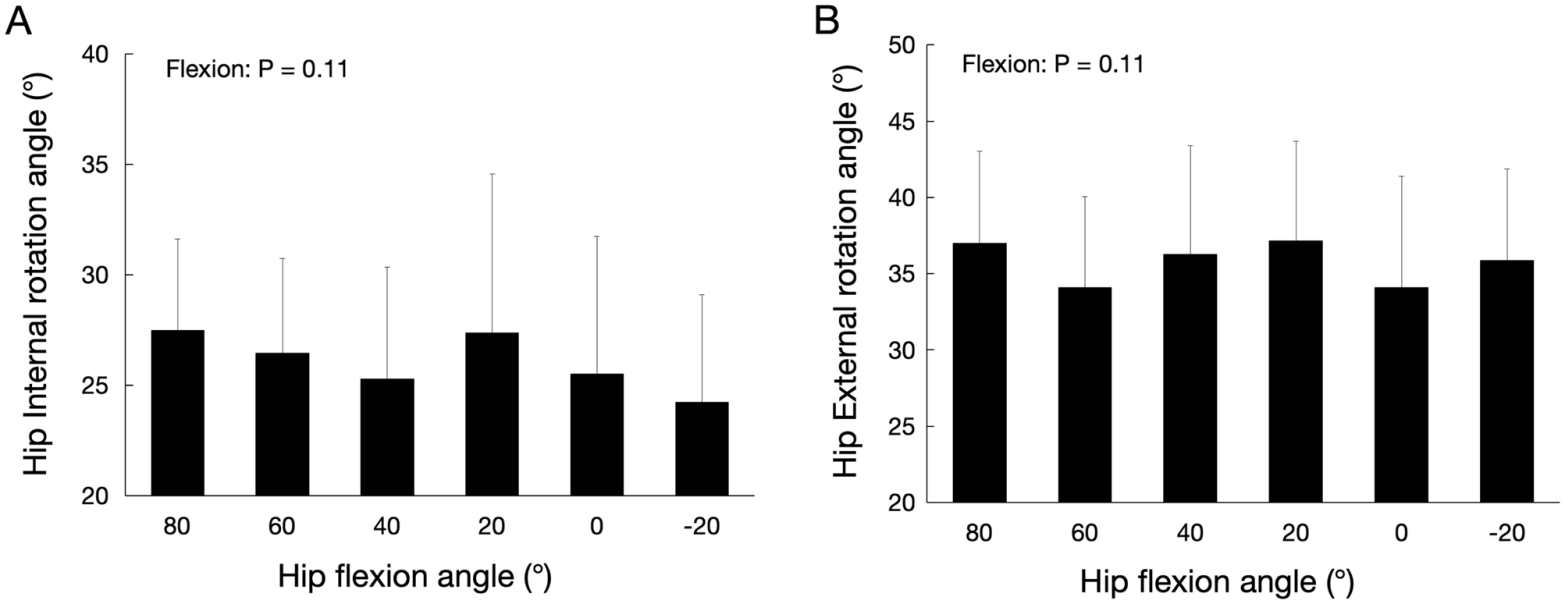
Maximum hip internal (**A**) and external (**B**) rotation angles at 40° hip abduction for each hip flexion angle.

Figure 11 shows the maximum hip abduction angle for each hip flexion angle. For the maximum hip abduction angle, a significant flexion effect was observed (F = 12.61, *P* < 0.01). The maximum hip abduction angle at the 20° hip extension was significantly lower than that at all hip flexion angles. Figure 12 shows the maximum hip internal and external rotation angles at the 40° hip abduction for each hip flexion. No significant flexion effect was observed for maximal hip internal and external rotation angles (internal rotation angle: F = 1.86; *P* = 0.11, external rotation angle: F = 1.86; *P* = 0.11).

## Discussion

The major findings of the present study were as follows: (1) increasing the shear modulus of the AL muscle associated with hip abduction depends on the hip flexion angle, and increasing that associated with hip abduction is higher at 20° hip extension than at 80° hip flexion; (2) changes in the shear modulus of the AL muscle associated with hip rotation depend on the hip flexion angle, and the shear modulus of the AL muscle increases by hip internal rotation only at 20° of hip extension regardless of the hip abduction angle.

The shear modulus of the AL muscle measured by SWE during elongation has a strong linear relationship with the passive force^16^. Similarly, previous studies have reported that this relationship is strongly linear in animal muscles^23,25^ and the human rectus femoris muscle^22^. In this study, the %RMS values of the AL muscle during the hip abduction and rotation tasks were 1.4 and 1.7%, respectively. Moreover, these observations were comparable to the %RMS of the rectus femoris muscle^26^ (1.3%) and hamstring muscles^27^ (1.4%) previously reported by studies that examined the changes in shear modulus during passive stretching. Therefore, changes in the shear modulus of the AL muscle may reflect the passive force of the AL muscle with elongation.

The increase in the shear modulus of the AL muscle associated with hip abduction depends on the hip flexion angle. Moreover, there is an insignificant difference between the shear moduli of the AL muscle for 20° hip extension and 80° hip flexion at 0° hip abduction, whereas the shear modulus of the AL muscle at 20° hip extension is higher than that at 80° hip flexion (approximately 2.7 times) at 40° hip abduction. Anatomically, the AL muscle has a hip flexion moment arm at the 0° hip flexion, and it increases with the hip extension because the AL muscle rises to the inferior pubic ramus and lodges into the middle third of the medial rip of the linea aspera^19,28^. Owing to this anatomical feature, AL muscle elongation is associated with hip extension, and the shear modulus of the AL muscle increases. Previous studies have already shown that the shear modulus of the AL muscle increases with the hip extension at 0° hip abduction^17^. Therefore, the increase in the shear modulus of the AL muscle during hip abduction is higher in the hip-extended position than in the hip-flexed position because the AL muscle is elongated in the hip-extended position. The maximum hip abduction angle at 20° hip extension angle is significantly lower than that at other hip flexion angles, whereas the shear modulus of the AL muscle at 20° hip extension is significantly higher than that at 40 and 80° hip flexions in the maximum hip abduction position. This result indicates that the passive force on the AL muscle in the hip-extended position is higher than that in the hip-flexed position in the maximum hip abduction position, even if the maximum hip abduction angle in the hip-extended position is lower than that in the hip-flexed position. However, the results of the present study differ from those of a previous study that shows that the shear modulus of the AL muscle does not depend on the hip flexion angle in the maximum hip abduction position^18^. The reason for this difference is that the previous study did not consider the pelvis of the subject, and there might have been a muscle contraction effect on the shear modulus because the EMG of the AL muscle was not measured during the shear modulus measurement^18^.

The shear modulus of the AL muscle associated with hip rotation also depends on the hip flexion angle, and it is significantly higher at 20° hip internal rotation than at 0° hip rotation and 20° hip external rotation at 20° hip extension. It is unknown whether the AL muscle has a hip internal or external rotation moment arm in the anatomical position. Dostal et al.^19^ reported that the AL muscle had a hip internal rotation moment arm in the anatomical position. In contrast, Leighton^29^ concluded that the AL muscle undergoes external hip rotation. In this study, it was unclear whether the AL muscle had a hip internal or external rotation moment arm because the hip rotation moment arm of each subject was not measured. However, a previous study showed a strong linear relationship between the shear modulus of the AL muscle and the passive force^16^. At 20° hip extension, therefore, there might be a passive force in the AL muscle due to the stretching of the AL muscle by hip internal rotation. At the maximum hip internal and external rotation angles at 40° hip abduction, the shear modulus of the AL muscle depends on the hip flexion angle, and the shear modulus of the AL muscle at 20° hip extension is significantly higher than that at 80° hip flexion. These results suggest that the passive force of the AL muscle may be higher at the hip-extended position than at the hip-flexed position for a 40° hip abduction angle.

The shear modulus of the AL muscle during hip abduction was found to be correlated with the passive hip adduction torque significantly, regardless of the hip flexion angle (r = 0.363–0.503, *P* < 0.01). The correlation coefficient is the highest at 80° hip flexion (r = 0.503) and the lowest at 20° hip extension (r = 0.363). Despite the moderate correlation, these results indicate that the shear modulus and passive hip adduction torque are more correlated in the hip-flexed position than in the extended position. Based on this, the passive hip adduction torque may include the stiffness of some muscles, joint capsules, and ligament tissues. Moreover, the effect of the stiffness of other tissues, except the AL muscle, on the passive hip adductor torque may be higher at the 20° hip extension than at 80° hip flexion.

In football, the AL muscle may get injured during kicking and change its direction ^6^. However, changes in the passive force of the AL muscle during these motions are unclear. This study demonstrated that the shear modulus of the AL muscle increases through hip abduction, extension, and internal rotation. We have already demonstrated a strongly linear relationship between the shear modulus and passive force in the AL muscle^16^. Therefore, the results of the present study indicate that the passive force of the AL muscle can be increased by hip abduction, extension, and internal rotation. This finding may provide useful information for elucidating the mechanism of AL muscle injury. Some previous studies reported that the hip abduction stretching improves the flexibility of the hip adductor muscles, which increases the maximal hip abduction angle^30–32^; however, specific stretching techniques for the AL muscle have not been developed. The findings of this study may provide insights into AL-specific stretching techniques.

The study had certain limitations. First, we measured the shear modulus of the AL muscle only at the proximal site because the AL muscle located on the surface of the thigh was the only proximal part. Owing to the technical limitations of SWE, it was difficult to measure the shear modulus of the deep layer because shear waves generated by push pulses are unlikely to occur. However, AL muscle injuries frequently occur in the proximal part^33^. Even if the shear modulus measurement site of the AL muscle was only proximal, the results of this study can be considered clinically relevant. Second, because we measured the shear modulus of the AL muscle at the hip positions, which is a combination of different directions of hip movements, each data point was measured at each rest hip position and not a passive dynamic movement. Thus, changes in the shear modulus associated with those in the hip joint angle in this study may differ from the changes in the shear modulus of the AL muscle that occur during passive dynamic movement. Third, it is unclear whether the changes in the shear modulus of the AL muscle are specific compared to other hip adductor muscles because we measured only the shear modulus of the AL muscle only at the knee-flexed position in this study. Because the gracilis muscle, which is a biarticular muscle, is stretched in the knee-extended position, the changes in the shear modulus of the AL muscle in the knee-extended position may be different from those in the knee-flexed position. To better understand the mechanical properties of the AL muscle, further studies are needed that compare the changes in the shear modulus of the other adductor with those of the AL muscle and measure the shear modulus of the AL muscle in the knee-flexed position. Lastly, the subjects in the present study were limited to males because there was no consensus on the effect of sex differences on the shear modulus^34–36^. However, there is a difference in the femoral bone morphology of different sexes^37^, which may be a confounding factor. Therefore, the changes in the shear modulus of the AL muscle associated with the hip joint angle in females may be different from those in males.

## Conclusion

In conclusion, this study demonstrated that the shear modulus of the AL muscle associated with hip abduction and rotation depends on the hip flexion angle. Specifically, the increase in the shear modulus of the AL muscle associated with hip abduction was higher in the hip extension position than in the hip flexion position. Moreover, the shear modulus of the AL muscle associated with hip rotation is independent of the hip abduction position and increased due to hip internal rotation only in the hip extension position. The results of the present study indicate that the passive force of the AL muscle may increase by not only hip abduction but also hip extension and internal rotation. This finding could be useful in elucidating the mechanism of AL muscle injury and developing AL-specific strength training or stretching techniques.

## Data Availability

The datasets generated during the current study are available from the corresponding author on reasonable request.
